# Out-of-Municipality Long-Term Care Insurance Benefits and Public Hospital Revenue Patterns in the Remote Oki Islands, Japan: An Exploratory Ecological Study

**DOI:** 10.7759/cureus.112668

**Published:** 2026-07-14

**Authors:** Kota Sakaguchi, Daisuke Horiuchi, Nobuyuki Ueno, Makoto Kaneko, Yoshihiko Shiraishi, Takashi Watari

**Affiliations:** 1 General Medicine, Shimane Prefectural Central Hospital, Shimane, JPN; 2 Faculty of Medicine, Shimane University, Izumo, JPN; 3 General Medicine, Gotsu Hospital, Gotsu, JPN; 4 General Medicine, Kitasato University School of Medicine, Sagamihara, JPN; 5 Comprehensive Community Medicine, Kitasato University School of Medicine, Sagamihara, JPN; 6 General Medicine Center, Shimane University Hospital, Izumo, JPN; 7 Integrated Clinical Education Center, Kyoto University Hospital, Kyoto, JPN

**Keywords:** ecological study, japan, long-term care insurance, public hospitals, rural health services

## Abstract

Background

Population aging and depopulation pose substantial challenges to the sustainability of healthcare and long-term care systems in rural and remote communities. This study aimed to describe temporal trends in out-of-municipality long-term care insurance (LTCI) benefits and contemporaneous public hospital management indicators in two remote island areas in Japan.

Methods

We conducted an exploratory descriptive ecological study using aggregated municipality-level LTCI and population data and hospital-level management data from fiscal year (FY) 2015 to FY2023 in the Oki Islands, Shimane Prefecture, Japan. We described trends in the Dozen region and Okinoshima Town, including population structure, the proportion of out-of-municipality LTCI benefits, inpatient and outpatient revenue, bed occupancy rates, and financial indicators. Percentage changes, annualized percentage changes, and outpatient-to-inpatient revenue ratios were calculated descriptively. No inferential hypothesis testing was performed.

Results

The population decreased in both areas. In the Dozen region, the proportion of out-of-municipality LTCI benefits increased overall from 9.5% in FY2015 to 21.9% in FY2023, whereas it remained relatively stable in Okinoshima Town, ranging from 2.6% to 3.2%. In Dozen Hospital, inpatient revenue decreased from JPY 330.8 million to JPY 260.6 million (-21.2%), while outpatient revenue increased from JPY 226.0 million to JPY 267.9 million (+18.6%). In Oki Hospital, inpatient revenue decreased from JPY 1,294.3 million to JPY 1,082.5 million (-16.4%), while outpatient revenue increased from JPY 1,035.5 million to JPY 1,116.2 million (+7.8%). The outpatient-to-inpatient revenue ratio increased from 0.68 to 1.03 in Dozen Hospital and from 0.80 to 1.03 in Oki Hospital.

Conclusions

The increase in out-of-municipality LTCI benefits in the Dozen region coincided with a comparatively large shift in the composition of Dozen Hospital revenue from inpatient toward outpatient services. These hypothesis-generating ecological findings do not demonstrate individual relocation or establish a causal relationship. Out-of-municipality LTCI benefits may warrant further evaluation as an administrative indicator alongside individual-level care utilization, residential histories, service capacity, and patient-flow data before being used to inform health policy or resource allocation.

## Introduction

Population aging and depopulation create substantial challenges for maintaining healthcare and long-term care services in rural and remote communities. Remote islands may be particularly vulnerable because geographic isolation, small populations, and uneven distributions of medical and long-term care resources can restrict access to care and affect the sustainability of local healthcare institutions [1-4].

Japan’s public long-term care insurance (LTCI) system was introduced to support the independence of older adults and reduce the burden on family caregivers [5]. The system is administered by municipalities, special wards, and extended associations across municipalities, which serve as insurers [6]. LTCI benefits may be paid for services delivered outside the insured person’s municipality of residence. Therefore, the proportion of out-of-municipality LTCI benefits may provide an administrative indication of the extent to which residents use long-term care services outside their municipality. However, this indicator does not directly measure residential relocation.

The availability and utilization of medical and long-term care services differ across regions in Japan [3,7]. One possible reason for receiving long-term care services outside the municipality is care-related relocation. In Japan, care-dependent older adults may move closer to their children or other family members to obtain support, a pattern sometimes described as “yobiyose” caregiving [8]. Frail older adults frequently have multimorbidity and functional impairment and may require substantial inpatient and long-term care services [9]. Nevertheless, out-of-municipality LTCI benefits may also reflect placement in a facility outside the municipality, temporary use of services near family members, or limited availability of particular services within the municipality.

At the health-system level, greater use of long-term care services outside a municipality may coincide with changes in the number and characteristics of care-dependent older adults remaining locally, available discharge destinations, and the local supply of long-term care services. These changes may, in turn, be accompanied by changes in demand for inpatient and outpatient hospital services and in the composition of hospital revenue. Conversely, changes in local hospital or long-term care capacity may influence the use of services outside the municipality. Accordingly, parallel temporal patterns between out-of-municipality LTCI benefits and hospital indicators should be regarded as hypothesis-generating rather than evidence of a directional or causal relationship.

Previous research has documented the effects of depopulation on population geography, rural disparities in healthcare access, and regional differences in medical and long-term care resources [1-4]. However, few studies have jointly examined municipality-level patterns of out-of-municipality LTCI benefits and public hospital management indicators in remote island settings.

The primary objective of this study was to describe temporal trends in the proportion of out-of-municipality LTCI benefits in the Dozen region and Okinoshima Town from FY2015 to FY2023. The secondary objective was to describe contemporaneous trends in public hospital management indicators in the two areas and to compare these patterns descriptively, without investigating or inferring a causal relationship.

## Materials and methods

Study design, setting, and period

**Figure 1:**
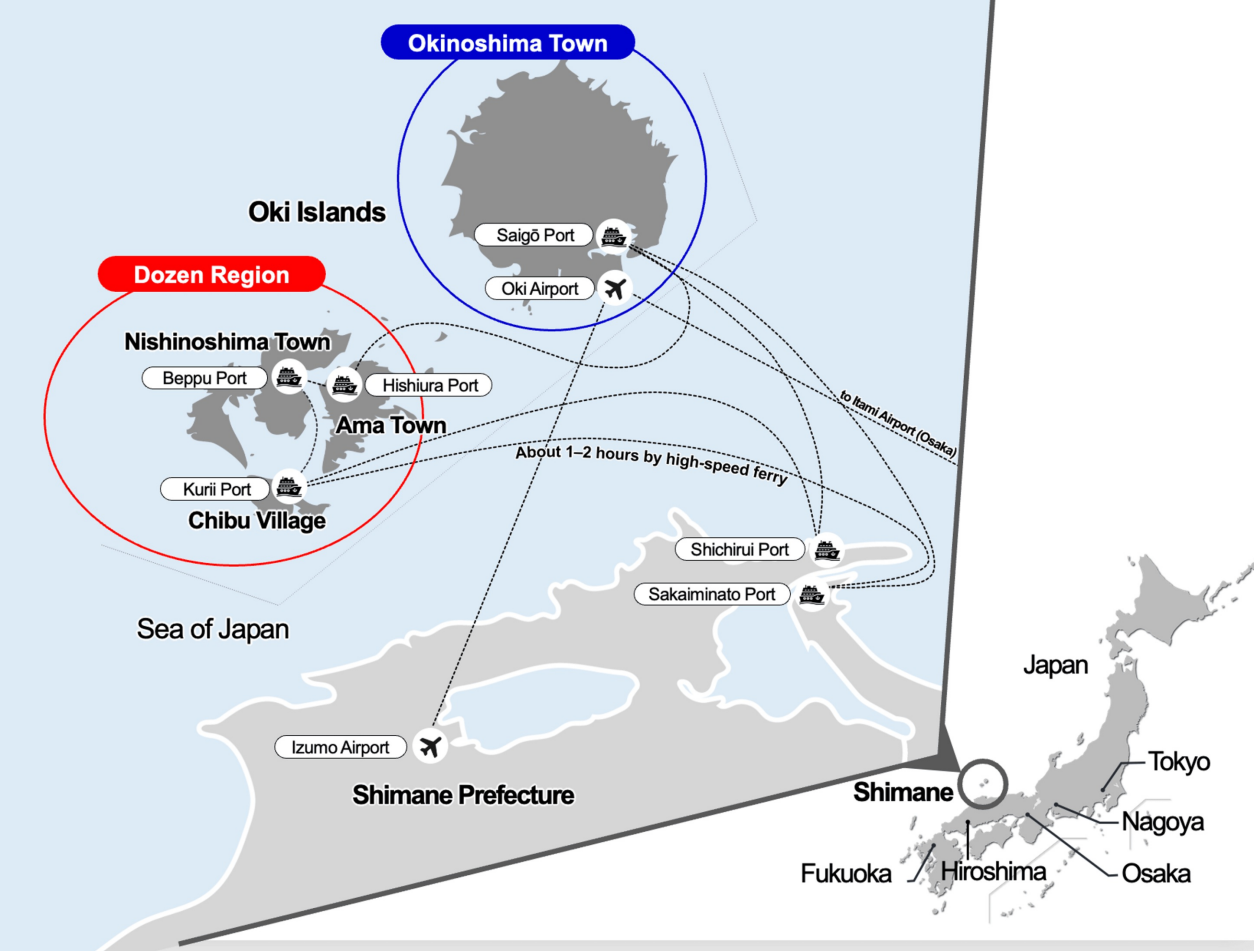
Map of the study setting in the Oki Islands, Shimane Prefecture, Japan. The Dozen region, comprising Nishinoshima Town, Ama Town, and Chibu Village, was designated as the primary study area, and Okinoshima Town was included as the comparison area. Both are geographically isolated island communities in the Sea of Japan. The figure was created by the authors.

We conducted an exploratory descriptive ecological study in the Oki Islands, Shimane Prefecture, Japan, a group of geographically isolated inhabited islands in the Sea of Japan (Figure 1).

The Dozen region, comprising Ama Town, Nishinoshima Town, and Chibu Village, was designated as the primary study area. Oki Dozen Hospital, hereafter referred to as Dozen Hospital, is the only hospital in the Dozen region and serves as the principal inpatient care provider for the three municipalities. Okinoshima Town is the largest municipality in the Oki Islands, and Oki Hospital is its principal public hospital.

Okinoshima Town was selected as the comparison area because it constitutes the other major inhabited island area within the Oki Islands and is also served by a public core hospital. The two areas therefore share a remote-island setting, the same prefectural context, and a broadly common administrative environment. However, Okinoshima Town was not intended to serve as a matched control. The two areas differ in population size, healthcare and long-term care resources, and the functional roles of their core hospitals. Comparisons were therefore interpreted as contextual and descriptive rather than causal.

The study period extended from FY2015 to FY2023, for which consistent municipality-level LTCI, population, and hospital management data were available. In Japan, the fiscal year runs from April 1 to March 31 of the following year.

Data sources and variables

LTCI Data

LTCI data were obtained from the Oki Wide Area Union, which administers the LTCI system in the Oki Islands. Aggregated municipality-level annual data were provided for research purposes. The dataset is not publicly available. Any request for access would be subject to permission from the Oki Wide Area Union and the relevant municipalities.

For each fiscal year, we extracted the amount of out-of-municipality LTCI benefits, defined as benefits paid for services delivered outside the municipality of residence, and the total amount of LTCI benefits. The denominator comprised the total LTCI benefit amount for each municipality as recorded in the annual aggregated dataset provided by the Oki Wide Area Union. Individual benefit categories were not available separately in the dataset used for this study.

The proportion of out-of-municipality LTCI benefits was calculated as follows:

$\left(\frac{\text{Out-of-municipality LTCI benefits}}{\text{Total LTCI benefits}}\right) \times 100$

This indicator was not interpreted as a direct measure of individual relocation. Rather, it was used as a descriptive administrative indicator of long-term care services utilized outside the insured municipality.

Population Data

Population data were obtained from official resident registry statistics provided by each municipality. We extracted the annual total population, population aged ≥65 years, and aging rate. The aging rate was calculated as the population aged ≥65 years divided by the total population and multiplied by 100.

Hospital Management Data

Hospital management data were obtained from the publicly available Local Public Enterprise Yearbook published by the Ministry of Internal Affairs and Communications, Japan [10].

We extracted annual data for Dozen Hospital and Oki Hospital, including inpatient revenue, outpatient revenue, bed occupancy rate, operating profit ratio, and ordinary profit ratio. Inpatient and outpatient revenues were used according to the corresponding accounting categories reported in the Yearbook, without reclassification. The same data source and category definitions were used for both hospitals throughout the study period.

Revenue values were retained in nominal Japanese yen because the objective was to describe the accounting values reported in the Yearbook. They were not adjusted for inflation. Consequently, changes in revenue should not be interpreted as equivalent changes in real purchasing power, service volume, or intensity of care.

Statistical analysis

The analysis was descriptive. Extracted annual values were cross-checked against the original source tables, and calculated proportions and percentage changes were recalculated before analysis. No missing annual values were identified, and no imputation was performed.

Annual trends in demographic indicators and out-of-municipality LTCI benefits were summarized for the Dozen region and Okinoshima Town from FY2015 to FY2023. Hospital management indicators were summarized for Dozen Hospital and Oki Hospital over the same period.

Percentage changes from FY2015 to FY2023 were calculated for inpatient and outpatient revenue. Annualized percentage changes were calculated as follows:

$\left[\left(\frac{\text{Value in FY2023}}{\text{Value in FY2015}}\right)^{\frac{1}{8}} - 1\right] \times 100$

To quantify the relative shift in revenue composition, we calculated the outpatient-to-inpatient revenue ratio for each hospital. Because bed occupancy rates and profit ratios were already expressed as percentages, their changes were reported in percentage points.

Correlation coefficients were not calculated because the study included only nine annual observations and shared secular trends could produce unstable or misleading correlations. Comparisons between the two areas were descriptive. No formal statistical hypothesis testing, regression modeling, or interrupted time-series analysis was performed.

Ethical considerations

This study used aggregated municipality-level and hospital-level administrative data and did not involve human participants, identifiable personal information, or biological specimens. Permission to use the aggregated LTCI data was obtained from the Oki Wide Area Union and the relevant municipalities. Under the Japanese Ethical Guidelines for Medical and Biological Research Involving Human Subjects, institutional ethical review and informed consent were not required.

## Results

Demographic trends and out-of-municipality LTCI benefits

Table 1 summarizes demographic trends and out-of-municipality LTCI benefits in the Dozen region and Okinoshima Town from FY2015 to FY2023. The total population decreased from 5,808 to 5,499 in the Dozen region and from 14,413 to 12,977 in Okinoshima Town. Although the overall trend in the Dozen region was downward, the source data showed an increase from 5,808 persons in FY2015 to 5,913 persons in FY2016, corresponding to 105 persons or 1.8%. The available aggregated data did not indicate whether this year-to-year change represented demographic fluctuation or an administrative revision; therefore, the reported values were retained without modification.

**Table 1:** Demographic trends and out-of-municipality LTCI benefits in the Dozen region and Okinoshima Town, FY2015-FY2023 FY: fiscal year (April 1 to March 31, of the following year); LTCI: long-term care insurance

Parameter	2015	2016	2017	2018	2019	2020	2021	2022	2023
Dozen region									
Total population (persons)	5,808	5,913	5,847	5,840	5,757	5,694	5,656	5,581	5,499
Population aged ≥65 years (persons)	2,499	2,487	2,485	2,498	2,488	2,511	2,460	2,415	2,387
Aging rate (%)	43	42.1	42.5	42.8	43.2	44.1	43.5	43.3	43.4
Out-of-municipality LTCI benefits (thousand JPY)	72,204	81,490	81,761	91,988	82,042	92,994	105,635	133,185	157,013
Proportion of out-of-municipality benefits to total LTCI benefits (%)	9.5	11.2	11.3	12.5	11.6	12.6	14.2	17.7	21.9
Okinoshima Town									
Total population (persons)	14,413	14,480	14,279	14,109	13,874	13,664	13,283	13,164	12,977
Population aged ≥65 years (persons)	5,527	5,636	5,641	5,644	5,620	5,603	5,569	5,580	5,620
Aging rate (%)	38.4	38.9	39.5	40	40.5	41	41.9	42.3	42.5
Out-of-municipality LTCI benefits (thousand JPY)	61,901	65,223	60,614	55,824	60,837	58,328	64,745	60,068	70,196
Proportion of out-of-municipality benefits to total LTCI benefits (%)	2.7	2.9	2.8	2.6	2.8	2.6	2.9	2.7	3.2

The aging rate remained high and relatively stable in the Dozen region, increasing slightly from 43.0% to 43.4%. In Okinoshima Town, the aging rate increased from 38.4% to 42.5%.

**Figure 2:**
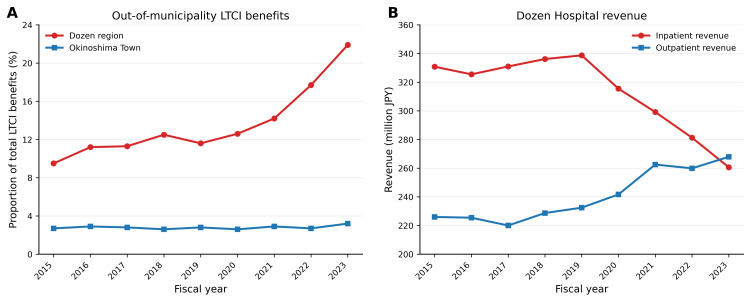
Trends in out-of-municipality LTCI benefits and Dozen Hospital revenue, FY2015-FY2023 (A) Proportion of out-of-municipality LTCI benefits among total LTCI benefits in the Dozen region and Okinoshima Town. (B) Inpatient and outpatient revenue trends in Dozen Hospital. FY: fiscal year (April 1 to March 31, of the following year); LTCI: long-term care insurance

The trends in out-of-municipality LTCI benefits differed between the two areas. In the Dozen region, the proportion increased overall from 9.5% in FY2015 to 21.9% in FY2023. In Okinoshima Town, the proportion remained relatively low and stable, ranging from 2.6% to 3.2% throughout the study period. Figure 2A illustrates the divergence in the proportion of out-of-municipality LTCI benefits between the Dozen region and Okinoshima Town.

Hospital management indicators

Table 2 summarizes the major hospital management indicators for Dozen Hospital and Oki Hospital from FY2015 to FY2023.

**Table 2:** Public hospital management indicators for Dozen Hospital and Oki Hospital, FY2015-FY2023 FY = fiscal year (April 1 to March 31, of the following year). All financial data were obtained from the Local Public Enterprise Yearbook that is published by the Ministry of Internal Affairs and Communications, Japan [10].

Parameter	2015	2016	2017	2018	2019	2020	2021	2022	2023
Dozen Hospital									
Operating profit ratio (%)	79.6	76.8	80.6	78	78.6	76.2	73.1	68.7	67.8
Ordinary profit ratio (%)	96.7	98.5	107.1	102.3	110.6	110.7	101.8	95.9	96.7
Bed occupancy rate (general) (%)	94.9	94.5	94.3	96.3	95.9	86.4	85.3	74.7	78.8
Bed occupancy rate (total) (%)	88.2	85.9	85.5	86.6	88.1	80.4	76.2	68.1	63.3
Inpatient revenue (thousand JPY)	330,806	325,470	330,972	336,167	338,711	315,467	299,182	281,260	260,615
Outpatient revenue (thousand JPY)	225,964	225,446	220,012	228,680	232,440	241,671	262,525	259,880	267,946
Oki Hospital									
Operating profit ratio (%)	85.6	79.6	79.5	81.3	79.9	74.5	72.8	70.7	69.1
Ordinary profit ratio (%)	102.3	97.9	96.9	100	101.8	105.1	107.1	108	101.1
Bed occupancy rate (general) (%)	84.2	77.2	82.1	84.2	76.9	64.4	64.1	63.2	65.8
Inpatient revenue (thousand JPY)	1,294,268	1,159,122	1,198,832	1,231,966	1,168,447	1,049,185	1,070,034	1,038,235	1,082,463
Outpatient revenue (thousand JPY)	1,035,455	1,013,196	1,012,143	1,043,194	1,094,504	1,053,448	1,070,905	1,095,468	1,116,212

In Dozen Hospital, inpatient revenue decreased from JPY 330.8 million in FY2015 to JPY 260.6 million in FY2023, representing a 21.2% decrease and an annualized decrease of 2.9%. In contrast, outpatient revenue increased from JPY 226.0 million to JPY 267.9 million, representing an 18.6% increase and an annualized increase of 2.2%. The outpatient-to-inpatient revenue ratio increased from 0.68 to 1.03, representing a relative increase of 50.5%.

In Oki Hospital, inpatient revenue decreased from JPY 1,294.3 million in FY2015 to JPY 1,082.5 million in FY2023, representing a 16.4% decrease and an annualized decrease of 2.2%. Outpatient revenue increased from JPY 1,035.5 million to JPY 1,116.2 million, representing a 7.8% increase and an annualized increase of 0.9%. The outpatient-to-inpatient revenue ratio increased from 0.80 to 1.03, representing a relative increase of 28.9%.

Other hospital management indicators also changed during the study period. In Dozen Hospital, the operating profit ratio decreased by 11.8%, the ordinary profit ratio was unchanged, the general-bed occupancy rate decreased by 16.1%, and the total-bed occupancy rate decreased by 24.9%. In Oki Hospital, the operating profit ratio decreased by 16.5%, the ordinary profit ratio decreased by 1.2%, and the general-bed occupancy rate decreased by 18.4%.

Figure 2B illustrates the divergence between inpatient and outpatient revenue trends in Dozen Hospital. The corresponding trends for Oki Hospital are presented in Table 2.

## Discussion

This exploratory ecological study described temporal patterns in out-of-municipality LTCI benefits and public hospital management indicators in two remote island areas. The proportion of out-of-municipality LTCI benefits increased substantially in the Dozen region but remained relatively low and stable in Okinoshima Town.

Inpatient revenue decreased, and outpatient revenue increased in both hospitals. The outpatient-to-inpatient revenue ratio increased from 0.68 in FY2015 to 1.03 in FY2023 in Dozen Hospital, representing a relative increase of 50.5%, and from 0.80 to 1.03 in Oki Hospital, representing a relative increase of 28.9%. This descriptive measure provides a reproducible basis for the observation that the relative shift in revenue composition was larger in Dozen Hospital. Nevertheless, the findings represent parallel temporal patterns and do not establish that out-of-municipality LTCI use caused changes in hospital revenue.

Okinoshima Town provided a contextual comparison within the same remote-island and prefectural setting rather than a matched counterfactual. Differences in population size, healthcare and long-term care capacity, and the functional roles of the two hospitals may have contributed to the observed patterns and may limit direct comparisons between the two areas.

Out-of-municipality LTCI benefits may reflect several different circumstances, including residential relocation, placement in a long-term care facility outside the municipality, temporary use of services near family members, or the absence of particular services within the municipality. The administrative data used in this study did not allow these circumstances to be distinguished. The indicator should therefore not be regarded as a validated measure of care-related migration. Rather, it may provide a descriptive signal of the extent to which insured residents receive long-term care services outside their municipality.

Care-related relocation, including the pattern described in Japan as “yobiyose” caregiving, represents one possible explanation for out-of-municipality LTCI use [8]. Frail older adults frequently have multimorbidity and functional impairment and may have substantial inpatient and long-term care needs [9]. It is therefore plausible that changes in where care-dependent older adults receive long-term care could coincide with changes in demand for local hospital services. However, this interpretation remains hypothetical because the present study did not include individual-level residential histories, clinical characteristics, medical or LTCI claims, or longitudinal care trajectories.

The findings suggest that population size and aging rate alone may not fully describe changes occurring within local health and long-term care systems. In remote communities, monitoring out-of-municipality LTCI benefits together with hospital activity and financial indicators may provide additional descriptive information for service planning. However, the present study does not demonstrate that expanding local long-term care capacity would change hospital utilization or financial performance, nor do the findings alone justify a particular policy or resource-allocation decision.

The COVID-19 pandemic may also have influenced hospital utilization and revenue, particularly from FY2020 onward. Changes in care-seeking behavior, elective admissions, infection-control practices, staffing, and service delivery may have affected inpatient and outpatient activity. Pandemic-related policy and financial responses may also have influenced hospital financial indicators. Because the present study did not include patient volumes, service-level data, or an unaffected counterfactual, the independent contribution of the pandemic could not be determined.

This study has several limitations. First, because all analyses were based on aggregated municipality-level and hospital-level data, the study is susceptible to ecological fallacy. Area-level patterns cannot be assumed to apply to individual residents, and individual-level inferences regarding residential relocation, LTCI utilization, or hospital use cannot be made [11].

Second, the proportion of out-of-municipality LTCI benefits is not a validated measure of residential relocation and may reflect several forms of service use. Third, the study included only two island areas and two hospitals. The areas differed in population size, service capacity, long-term care resources, and hospital function; consequently, Okinoshima Town should be interpreted as a contextual comparison rather than a matched control.

Fourth, hospital revenue may have been influenced by unmeasured factors, including reimbursement revisions, staffing changes, service reorganization, the COVID-19 pandemic, and changes in service prices or intensity. Revenue indicators do not directly measure the number or clinical characteristics of patients receiving care.

Fifth, revenues were analyzed in nominal Japanese yen and were not adjusted for inflation. An increase in nominal revenue, therefore, does not necessarily indicate an increase in real revenue, service volume, or purchasing power. Sixth, the two public hospitals do not necessarily represent all healthcare utilization within their respective areas. These limitations restrict the generalizability and causal interpretation of the findings.

Future studies should combine individual-level LTCI and medical claims data, residential histories, hospital utilization data, and qualitative information regarding family decision-making. Multiregional studies are also needed to determine whether out-of-municipality LTCI benefits can serve as a reproducible indicator of external long-term care service use and whether such patterns are consistently associated with changes in rural hospital function.

## Conclusions

In the Dozen region, the proportion of out-of-municipality LTCI benefits increased substantially between FY2015 and FY2023. During the same period, both Dozen Hospital and Oki Hospital experienced declining inpatient revenue and increasing outpatient revenue, with a larger relative increase in the outpatient-to-inpatient revenue ratio at Dozen Hospital.

These findings are hypothesis-generating and do not establish individual relocation, causality, or a direct functional relationship between external long-term care use and hospital revenue. They therefore do not, by themselves, support a specific change in health policy or resource allocation. Nevertheless, out-of-municipality LTCI benefits may warrant further evaluation as an administrative signal to be monitored alongside service capacity, patient flows, residential histories, and individual-level medical and LTCI claims data in future research and regional health-system planning.
